# Androgen Elevation Accelerates Reproductive Senescence in Three-Spined Stickleback

**DOI:** 10.3389/fcell.2021.752352

**Published:** 2021-12-17

**Authors:** Mirre J. P. Simons, Marion Sebire, Simon Verhulst, Ton G. G. Groothuis

**Affiliations:** ^1^ School of Biosciences, University of Sheffield, Sheffield, United Kingdom; ^2^ The Centre for Environment, Fisheries and Aquaculture Science, Weymouth, United Kingdom; ^3^ Behavioural Biology, Centre for Behaviour and Neuroscience, University of Groningen, Groningen, Netherlands

**Keywords:** aging, fecundity, carotenoid, stickleback, signaling, sexual hormone, sexual selection

## Abstract

Costs of reproduction shape the life-history evolution of investment in current and future reproduction and thereby aging. Androgens have been proposed to regulate the physiology governing these investments. Furthermore, androgens are hypothesized to play a central role in carotenoid-dependent sexual signaling, regulating how much carotenoids are diverted to ornamentation and away from somatic maintenance, increasing oxidative stress, and accelerating aging. We investigated these relationships in male three-spined stickleback in which we elevated 11-ketotestosterone and supplied vitamin E, an antioxidant, in a 2 × 2 design. Androgen elevation shortened the time stickleback maintained reproductive activities. We suspect that this effect is caused by 11-ketotestosterone stimulating investment in current reproduction, but we detected no evidence for this in our measurements of reproductive effort: nest building, body composition, and breeding coloration. Carotenoid-dependent coloration was even slightly decreased by 11-ketotestosterone elevation and was left unaffected by vitamin E. Red coloration correlated with life expectancy and reproductive capacity in a quadratic manner, suggesting overinvestment of the individuals exhibiting the reddest bellies. In contrast, blue iris color showed a negative relationship with survival, suggesting physiological costs of producing this aspect of nuptial coloration. In conclusion, our results support the hypothesis that androgens regulate investment in current versus future reproduction, yet the precise mechanisms remain elusive. The quadratic relationships between sexual signal expression and aspects of quality have wider consequences for how we view sexual selection on ornamentation and its relationship with aging.

## Introduction

Organisms evolve to optimize allocation of resources between different physiological processes to maximize fitness. Such resource-based trade-offs are central to life-history theory (Stearns, 1989) and have been adopted throughout biology, including the biology of aging (Maklakov and Chapman, 2019). Questions concerning, for example, the optimal arrival time at breeding sites (Kokko, 1999), litter size (Dijkstra et al., 1990; Sikes et al., 1998), foraging effort (Abrams, 1991), dietary restriction (McCracken et al., 2020a), and prey choice (Rutten et al., 2006) can all be explained in a framework of fitness costs and benefits. A central or arguably ultimate cost in life history and the biology of aging is the cost of reproduction (Reznick et al., 2000; Lemaître et al., 2015). If there are no costs of producing offspring, why not simply produce more offspring to increase fitness?

The most direct test of these “costs of reproduction” is to increase reproductive effort experimentally and measure the long-term fitness consequences for the parents. In birds, this approach, by manipulating clutch or brood size, has been used many times. Yet, despite there being some undisputed demonstrations (Daan et al., 1990; Tinbergen and Daan, 1990) of costs of reproduction, a recent meta-analysis of 29 studies (Santos and Nakagawa, 2012) found that parental effort was associated with survival only in males, but not in females, with notably small overall effect sizes. Possibly, the fitness costs of parental effort are not traded off exclusively against future survival but also to future reproduction. Alternatively, the costs of an increased brood size are mainly paid by the offspring. This notion is supported by the finding that, in general, animals will not increase parental effort to such a degree that it fully compensates for the extra provisioning and care required by the offspring added, reducing offspring quality (Dijkstra et al., 1990; Simons et al., 2011). Indeed, the combined effects of brood enlargement studies on current reproduction, future reproduction, parental survival, and offspring fitness can reduce the total sum of fitness gained (Smith et al., 1989; Dijkstra et al., 1990; Tinbergen and Daan, 1990). Thus, whether the relationship between survival and investment in reproduction is causal and central in shaping the aging phenotype of animals remains less clear than current theory predicts.

In *Drosophila*, more suitable for artificial selection experiments than vertebrates, a genetic relationship between reproduction and survival has been demonstrated (Flatt, 2011). Surprisingly, however, there are multiple examples of long-lived mutants or experimental manipulations in *Drosophila* and *Caenorhabditis elegans* that extend lifespan without reduced or even increased fecundity (Flatt, 2011; McCracken et al., 2020b; Yamamoto et al., 2020; Lind et al., 2021). Note that these observations are made in the laboratory environment, and thus might not occur in the wild (Briga and Verhulst, 2015). Costs of reproduction therefore remain plausible but may be difficult to demonstrate or detect for different reasons. Physiological costs of reproduction may be context specific (Simons et al., 2014a), could be difficult to measure, or might be compensated or temporally dynamic.

Costs are also central to sexual signaling theory (Kotiaho, 2001; Számadó, 2011), and the cost of sexual traits for acquiring fertilizations should be viewed as part of the cost of reproduction (Höglund et al., 1998). According to the handicap hypothesis, mate choice for traits signaling male quality is only evolutionary stable when the trait bears costs, precluding cheating. Behavioral punishment (Számadó, 2011), energetic investment (Grafen, 1990; Candolin, 1999), mechanistic constraints (Emlen et al., 2012), and specific resource investment (Simons et al., 2014b) are mechanisms mediating the fitness cost of sexual signals. This diversity in the nature of costs may hamper detection of costs of sexual signals (Svensson and Wong, 2011), also because they may not be apparent in a laboratory setting where behavioral punishment is not possible, or because food is supplied *ad libitum*.

In general, all investment into reproduction, sexual signaling, and otherwise should be viewed as investment into current reproduction (Höglund et al., 1998), which is predicted to trade off with somatic maintenance and repair. It is this central trade-off that is persistently viewed as central in the biology of aging field, and forms the basis of the disposable soma theory of aging (Kirkwood, 2002). Investing in reproduction at the cost of somatic maintenance is usually optimal, because extrinsic mortality (mortality that cannot be fully intrinsically controlled) is almost never zero and investment into the soma is lost at death by an extrinsic cause (Williams, 1957). Any physiological investment that increases reproductive success is thus expected to reduce future reproduction either *via* accelerated mortality or *via* reproductive senescence.

Given this connection between any physiological cost paid to enhance current reproductive success at the expense of future reproductive success, we may expect physiological regulators balancing these investment decisions. Hormones, and in particular sex hormones, have gathered considerable attention in this regard (Hau, 2007; Regan et al., 2019; Bell, 2020; Garratt et al., 2020; Sugrue et al., 2021). In sexual signaling studies, testosterone specifically has attracted considerable attention for several reasons. First, the immunocompetence handicap hypothesis postulates that testosterone both suppresses the immune system and enhances expression of sexual traits and behavior (Folstad and Karter, 1992; Sheldon and Verhulst, 1996). Yet, evidence for direct immunosuppressive effects of testosterone is limited (Roberts et al., 2004), but see Newhouse and Vernasco (2020). In contrast, however, the reverse does hold, immune activation suppresses plasma testosterone, suggesting that testosterone plays a role in the trade-off between reproduction and somatic maintenance (Boonekamp et al., 2008). Second, testosterone has been shown to elevate carotenoid-based coloration (Blas et al., 2006; Kurtz et al., 2007; Perez-Rodriguez et al., 2008; Khalil et al., 2020), thereby possibly mediating the trade-off between current reproductive effort and oxidative stress (Schantz et al., 1999), induced by allocating carotenoids, an antioxidant [but see Koch et al. (2018) and Koch and Hill (2018)], away from maintenance towards sexual signaling (Peters, 2007; Svensson and Wong, 2011; Simons et al., 2012a). The link between oxidative stress and carotenoid signaling has gained further support by the increases in coloration observed when antioxidants are supplemented, such as vitamin E in stickleback (*Gasterosteus aculeatus*) (Pike et al., 2007a) and in gulls (*Larus michahellis*) (Pérez et al., 2008), but see Karu et al. (2008) and Giraudeau et al. (2013). A trade-off concerning oxidative stress can also link testosterone to immune suppression, given that higher levels of oxidative stress are hypothesized to negatively affect immunity (Kurtz et al., 2007; Peters, 2007; Costantini and Møller, 2009), but see Casagrande et al. (2012) and Newhouse and Vernasco (2020). Third, testosterone has also been suggested to increase metabolic rate but evidence is mixed (Buttemer et al., 2008; Holtmann et al., 2017), yet could possibly enhance food intake and thereby carotenoid intake or growth of bodily extremities used as ornaments (Mougeot et al., 2004).

These multiple relationships between testosterone, physiology, and sexual signaling hamper the interpretation of negative findings on the trade-off between current and future reproductive success as costs may come about in physiological aspects other than those under study. Moreover, which and how physiology is altered by elevated testosterone may vary over time or cost may come to expression only later in life. Long-term experiments of the physiological consequences of elevated testosterone are therefore required.

In several of such longer-term studies, testosterone has been shown to induce costs. Experimental elevation of testosterone in adult male brown-headed cowbirds (*Molothrus ater*) showed reduced return rates, and this has been explained by experiencing higher rates of aggression, because testosterone-implanted individuals also showed more signs of injury likely incurred during fighting (Dufty, 1989). Male testosterone-implanted mountain spiny lizards (*Sceloporus jarrovi*) also show reduced survival, but this effect is negated by food supplementation, suggesting an energetic cost (Marler and Moore, 1991). Experimental elevation in males of another lizard species (*Psammodromus algirus*) also reduced survival and increased ectoparasitic infestation (Salvador et al., 1996). In birds, survival of dark-eyed juncos (*Junco hyemalis*) (Reed et al., 2006), red grouse (*Lagopus lagopus scoticus*) (Moss et al., 1994; Mougeot et al., 2005; Redpath et al., 2006), and red-legged partridges (*Alectoris rufa*) (Alonso-Alvarez et al., 2020) was also lowered in individuals in which testosterone was experimentally elevated. Return rates of testosterone-implanted greater prairie-chicken cocks (*Tympanunchus cupido*) males were also lower although not significantly so (Augustine et al., 2011).

These studies suggest that testosterone elevation indeed has long-term survival costs. However, in several of these studies, elevations of testosterone were in the pharmacological range and/or were maintained after the breeding season in which the hormone is not elevated. So, to what extent elevated exposure to testosterone reduces survival or future reproduction under more natural conditions is not clear. In addition, effects on reproductive senescence and mechanistic links to carotenoid-dependent sexual signal expression were not directly investigated in these studies. Here, we test whether (11-keto)-testosterone (the most biologically active androgen in most teleost fish) modulates the trade-off between current and future reproduction in three-spined stickleback. Reproductive behaviors and sexual coloration are absent in castrated stickleback, but can readily be restored by 11-ketoandrostenedione, which is rapidly converted to 11-ketotestosterone (Borg and Mayer, 1995). Male sticklebacks produce nests from algae and plant material, glued together with “spiggin,” produced in their kidneys in response to 11-ketotestosterone (Jakobsson et al., 1996; Jakobsson et al., 1999). Using elaborate courtship, males attract gravid females to their nest to spawn and care for the offspring, and they repeat this nesting cycle multiple times in a single breeding season (Wootton and Robert, 1984). During their breeding season, stickleback exhibit a carotenoid-dependent trait, their reddish belly (Wedekind et al., 1998), which is subject to female choice (Künzler and Bakker, 2001; Pike et al., 2007b) and shows senescence (a decrease in functioning attributed to aging) within one breeding season (Kim et al., 2016). Males with redder bellies were previously found to have longer lifespans, and carotenoid supplementation extends lifespan and the time reproductive effort can be maintained (Pike et al., 2007b). Stickleback populations can either inhabit fresh water throughout the year or migrate to sea and back to breed in spring (anadromous populations).

The subjects of this study are wild-caught individuals on migration from sea to their breeding grounds. This population has been reported to be annual (van Mullem and van der Vlugt, 1964) (including more contemporary information from the Dutch Water Board). Size distribution data from caught stickleback in fishways, estuaries (including fish included in this study), and at the freshwater breeding grounds at the start of the breeding season showed a single peak in the distribution. Notably returning fish to sea also indicated a single peak of juvenile fish on their return migration (personal communication with and reports from the Dutch Water Board). Size density distributions are the most practical indication of the age distribution of populations of small fish. All indications from the limited data (from peer-reviewed sources and ecological reports) available on this population are therefore that the ecology of this population is an anadromous population that has an annual breeding lifecycle in freshwater. Hence, reproductive activities during a single breeding season likely determine lifetime reproductive effort. We hypothesize that 11-ketotestosterone elevation increases investment in current reproduction, e.g., nest building and sexual coloration, at the cost of maintaining the soma and thereby future reproduction.

## Methods

### Animals

Anadromous three-spined stickleback were caught using a lift net at the locks of Noordpolderzijl, the Netherlands (53°25′56″N, 6°34′ 59″E). Small leaks of fresh water through the locks attract stickleback into the estuary when they start migration toward fresh water early spring. Fish were transported to the laboratory (<25 km away) by car in aerated buckets filled with water from the estuary. In our aquarium facility, fresh water was added to adjust the fish to fresh water conditions across several days. Groups of fish were housed together in large glass aquaria (>60 × 30 × 30 cm, L × H × W).

### Setup

At the start of the experiment, individual males (*N* = 237) were housed in individual plastic tanks (27.5 × 17.5 × 17 cm, L × H × W, Ferplast geolarge), covered with a see-through plastic lid, containing a plastic plant (in the front of the tank, Tetra Plantastics Ambulia *Limnophila heterophylla*, 11–15 cm) and a pressure air (provided *via* connected tubing by a Resun LP-100 air-pump)-operated filter (at the back of the tank, Europet Bernina). One side adjacent to another tank was blinded with white adhesive plastic that precluded any visual contact between the fish. The tanks were set in eight vertically connected steel cabinets each containing six rows of shelves. Treatments were distributed across the cabinets balanced evenly for row and column and fish started the experiment distributed across 5 days balanced for treatment to divide the time required for husbandry and measurements. The room was air-conditioned to keep water temperatures at 18°C. Lighting, LD 16:8, corresponding to the daylength at the height of summer in the Netherlands, started when males were put in individual tanks, and was provided by fluorescent tubes (OSRAM Cool White, L40W/640SA) placed on the ceiling in front of the cabinets.

Fish were fed every morning with defrosted red bloodworms, (*Chironomus*, 3F Frozen Fish Food) in portions of ±0.25 g using a plastic pipette. If after 15 min of the first portion an individual fish had finished all the provided food, it received another portion, to achieve near *ad libitum* feeding without detrimental effects of overfeeding on water quality. At the end of each day (at least 1 h after the first feeding round), excess food was removed from each individual tank using a plastic syringe. To stimulate sexual behavior, gravid females in a plastic see-through jar were shown to individual males each day for 5 min. Each week, all water of each individual tank was changed, excluding the water retained in the small filter compartment.

Males were provided with 400 threads of green polyester threads (0.840 g of ±6-cm-long threads) (Barber, 2001; Rushbrook et al., 2008) placed behind the artificial plant and a petri dish (placed in the back of the aquarium) filled with white aquarium sand as nesting material. Each week, the fish received new nesting material and had to rebuild their nest. Nests were examined each day, and if completed (judged by the presence of a tunnel in the nest) and if all material was used in the nest, a portion of extra green polyester threads (0.150 g) was added to the aquarium behind the artificial plant. This protocol allowed us to calculate a metric of nesting intensity as a composite of nest material used and speed of nest completion. This metric was expressed as the amount of days nest material was added minus the days it took to complete the nest.

The ability to build nests we used as a proxy of reproductive capacity given that without a nest a male stickleback cannot produce offspring, when sneaking of fertilizations that sometimes happen in specific populations is ignored. Our experimental protocol therefore did not assess paternal care at any point. We purposefully studied the consequences of androgens on the reproductive phase in which males display and attract females, as we were interested in trade-offs with carotenoid-dependent signaling. The photoperiod was changed to a short photoperiod (LD 8:16) after 160 days, when less than 10% of the individuals were showing nest building behavior. At that point, nest material provision and female stimulation ceased and individuals were subsequently only monitored for survival.

### Injections and Circulating 11 kt Concentrations

After 1 week of acclimatization, individual males received two intraperitoneal injections (using 0.3-ml syringes, Becton Dickinson, Micro-Fine) of molten cocoa butter (14 μl/g fish body mass). The cocoa butter was injected at a temperature of 37°C (and solidified quickly in the fish kept in water of 18°C) and was loaded with 11-keto-androstenedione (in suspension, 4 mg/ml, based on a pilot study described below; Sigma Aldrich) and/or with vitamin E (dissolved, 226 mg/ml, α-tocopherol, Sigma Aldrich) or with nothing. With these combinations, we created a balanced 2 × 2 design of 11-keto-androstenedione and vitamin E. Our rationale for choosing vitamin E as a supplement was to study the suggested androgen-regulated relationship between carotenoid-dependent signaling and oxidative stress (Peters, 2007). To this end, we wanted to supplement an antioxidant, rather than pigmentary carotenoids with disputed antioxidant potential (Simons et al., 2012b), and vitamin E has previously been shown to increase the red belly of the stickleback (Pike et al., 2007a). In fish, 11-keto-androstenedione is rapidly converted to 11-ketotestosterone and similar methods have been used in three-spined stickleback previously to elevate 11-ketotestosterone concentrations (Páll et al., 2002; Kurtz et al., 2007), the main androgen in most male teleost fish (Borg et al., 1993; Borg, 1994; Borg and Mayer, 1995).

**FIGURE 1:**
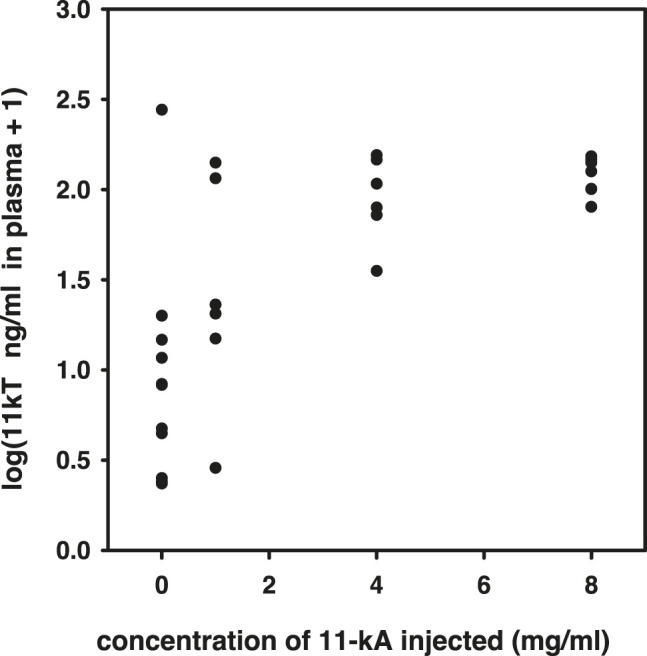
Results of the pilot experiment to determine resulting plasma concentrations of 11-ketotestosterone after treatment with different dosages of 11-keto-androstenedione.

Prior to the main experiment, but in the same spring, we carried out a pilot experiment to determine the appropriate dose of 11-keto-androstenedione. We injected, as described above, concentrations of 1, 4, and 8 mg/ml 11-keto-androstenedione and subsequently obtained plasma 5 days later. 11-Ketotestosterone levels in plasma were determined by radioimmunoassay (RIA) and were found to be elevated strongly with the 4 mg/ml dose, without any apparent further increase at the highest dose [0 vs. 4, *χ*(1)^2^ = 7.4, *p* = 0.007; 4 vs. 8, *χ*(1)^2^ = 0.62, *p* = 0.43; Figure 1].

At 2, 3, 4, and 5 weeks after the injections, random subsets of individuals of the main experiment were sacrificed for blood collection balanced for treatment. The fish were killed by a blow to the head, the tail was cut just posterior of the anus, and blood was collected from the caudal vein using heparinized glass capillaries. Blood was kept on ice until plasma was obtained *via* centrifugation (850 RCF for 7 min). Hematocrit of the individual samples was measured from the centrifuged capillaries with a digital caliper (Mitutoyo, to the nearest 10 μm). Plasma was stored in −80°C prior to analyses.

Individual fish were weighed prior to blood taking and, after blood taking, the mass of the testes, liver, kidney, and spleen were determined. 11-Keto testosterone levels were determined *via* radioimmunoassay (RIA) as previously described (Sebire et al., 2007). After thawing, the plasma samples were centrifuged at 13,000 rpm for 3 min at 4°C and then 5 μl was transferred into a 1.5-ml Eppendorf tube. Distilled water (100 μl) and ethyl acetate (1 ml) were added to the tube and vortexed for a few seconds. The samples were again centrifuged (13,000 rpm for 3 min at 4°C) and the bottom of the tube was subsequently placed briefly into liquid nitrogen. The organic phase was separated from the frozen aqueous phase into a glass tube. This extraction was repeated a second time. The ethyl acetate extracts were dried under a nitrogen stream at 45°C and redissolved in 500 μl of RIA buffer prior to analysis by RIA.

**FIGURE 2:**
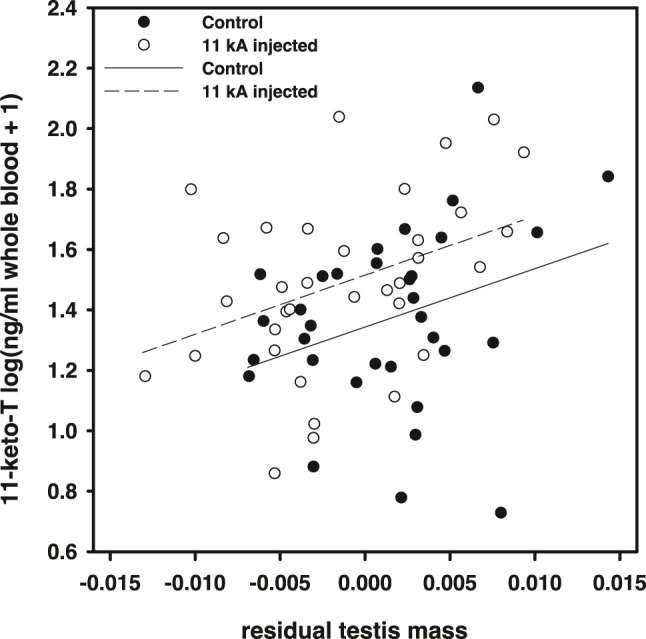
11-ketotestosterone plasma concentrations in the main experiment regressed against residual testis mass (against body mass). Open dots and dashed regression line, 11-keto-androstenedione treated individuals; closed dots and solid line, controls.

**FIGURE 3:**
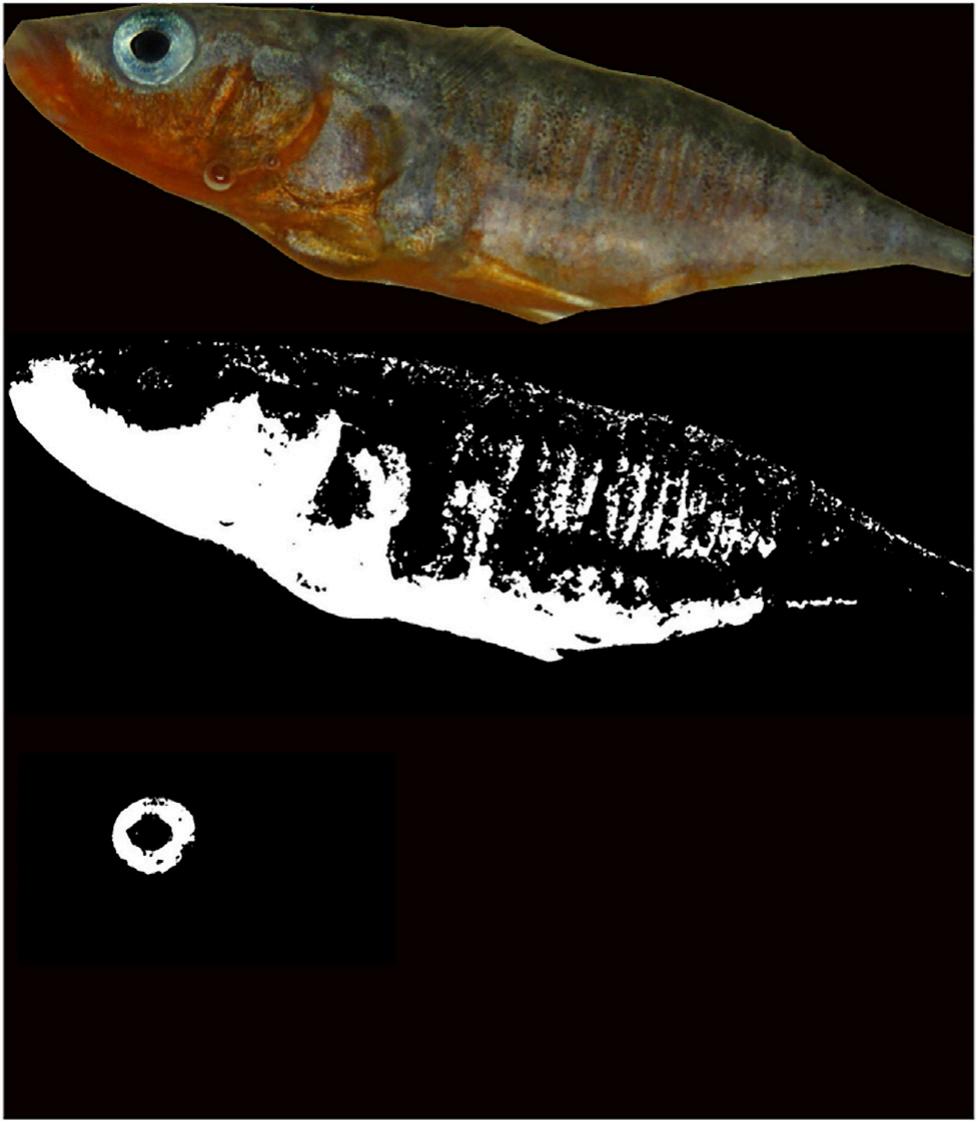
Example of the automatic selection and thresholding used to obtain red and blue chroma of the nuptial coloration of the stickleback. Top shows the individual stickleback extracted from the picture, the middle part shows the selected pixels (in white) above the red chroma threshold, and the bottom part shows the extraction of the blue iris, also using chroma thresholding.

Within the actual experiment, 11-keto-androstenedione treatment increased 11-ketotestosterone levels but relatively mildly [*F*(1,64) = 5.97, *p* = 0.017; Figure 3]. Note that these samples were taken later in the season (relative to the time of injection) than in the pilot study (Figure 1), which may explain the lower elevation measured. However, we did not detect an effect of time at which plasma was collected in this dataset [*F*(1,61) = 0.18, *p* = 0.91] which suggests that the mild elevation in 11-ketotestosterone our treatment induced was maintained for at least 5 weeks, yet levels prior to 2 weeks were probably higher, judging from the elevation measured in the pilot study. Furthermore, we only detected a significant effect of our treatment if we took natural variation of testis size into account, which covaried positively with 11-ketotestosterone plasma levels [*F*(1,64) = 9.20, *p* = 0.004; Figure 2]. Compared to the population variance in 11-ketotestosterone, independent of variance attributable to testis size, our experimental treatment with 11-keto-androstenedione resulted in an elevation of 0.63 standard deviation of 11-ketotestosterone. So, the 11-ketotestosterone level of a given fish in the 11-keto-androstenedione-treated group was elevated beyond its own endogenous production as determined by its testis size. This rationale is in line with the lack of feedback of circulating 11-ketotestosterone concentrations on its production in this species, implicating that experimental elevation of the hormone does not impact on its endogenous production. For example, removal of one testis halves 11-ketotestosterone levels (Hellqvist et al., 2002). No effects of vitamin E treatment on 11-ketotestosterone levels were detected (*p* > 0.77). Variation in 11-ketotestosterone did not correlate with maximum or average blue or red breeding coloration prior to sacrificing (*p* > 0.64 and *p* > 0.10, respectively).

### Breeding Coloration

Weekly measurements of coloration were made using digital photography (Sony α-200) with fixed camera setting and in a controlled lighting environment. Fish were placed in a small glass container with a piece of foam at the back to restrain the fish to the front of the glass with its body side. The glass was fitted within a holder to allow it to be placed in a fully darkened box slightly tilted to avoid reflections in the glass. The camera was attached to this setup and a lightproof fabric was wrapped around the camera to avoid any outside light entering the box. A white-LED ring light (Sony HVL-RLAM) was placed on the lens of the camera and lit the box.

As digital cameras do not respond linearly to light, and hence are biased in measuring properties of light reflectance (e.g., color) (Stevens et al., 2007; Pike, 2011), we calibrated (Stigell et al., 2007) our camera with a large set of color patches (Munsell Glossy Edition, with known reflectance from the Joensuu Spectral Database) under the same lighting and fixed camera settings. Such an approach allows for an accurate representation of reflectance spectra from RGB values extracted from digital pictures (Stigell et al., 2007; Simons et al., 2012c; Simons et al., 2016). Fish were extracted from the pictures automatically using thresholding, cluster analysis, and alpha shapes in Matlab. The reddest and bluish part (consisting largely of blue iris coloration) of the individual fish were selected using image segmentation *via* thresholding of chroma from the simulated spectra per pixel (Figure 3). If all pixels fell below this threshold, this data point was excluded from the analysis, which happened in 8% of the cases within the analyses of blue coloration and in 0% of the cases where we analyzed red pigmentation. From these selected patches, we calculated an average simulated spectra to estimate the chroma of the blue (summed reflectance between 420 and 540 nm divided by total reflectance between 420 and 740 nm) and red (summed reflectance between 620 and 740 nm divided by total reflectance between 420 and 740 nm) breeding coloration for each individual fish. The red and blue coloration, with the latter relatively understudied, of three-spined stickleback determine sexual attractiveness and are associated with aspects of quality (Milinski and Bakker, 1990; Künzler and Bakker, 2001; Rush et al., 2003; Pike et al., 2007b; Flamarique et al., 2013).

### Statistical Analyses

All individual measures on the fish were analyzed with general linear models or mixed models. If multiple measures from a fish were included, a random intercept for each fish was included in the model to correct for pseudo-replication. Note, the time variable (“week”) in the experiment was included as a categorical variable in the mixed models, as ignoring or misestimating random slopes in mixed models leads to a large inflation of type I error (Schielzeth and Forstmeier, 2009). Interpretation of patterns over time were based on least square means and their associated standard errors as provided in the figures. Model selection was based on backward selection of a model containing the interactions of the two treatment variables with the week of the experiment and mass at the start of the experiment. Breeding coloration was solely analyzed in fish that were still breeding (buildings nests), because breeding coloration fades quickly after the termination of reproductive activities. Reproductive senescence, as measured by the period that reproductive activities (i.e., nest building) were maintained, was analyzed using time to event Cox proportional hazard models (“coxph”) (Therneau and Grambsch, 2000). These models used right-hand censoring for the sacrificed animals, those that died an accidental death, or those that were still building nests when the experiment was terminated (21 weeks after the injections). All analyses were performed in SAS JMP 7.0 and R. Sample sizes differ between the analyses that span across the breeding season, due to a decline in the number of eligible fish (i.e., surviving and in breeding state). Hence, degrees of freedom of each analysis provide information on the underlying sample size. No violations of the assumption of a Gaussian distribution were detected in the dependent variables and residuals of the parametric models.

## Results

### Reproductive and Mortality Senescence

**FIGURE 4:**
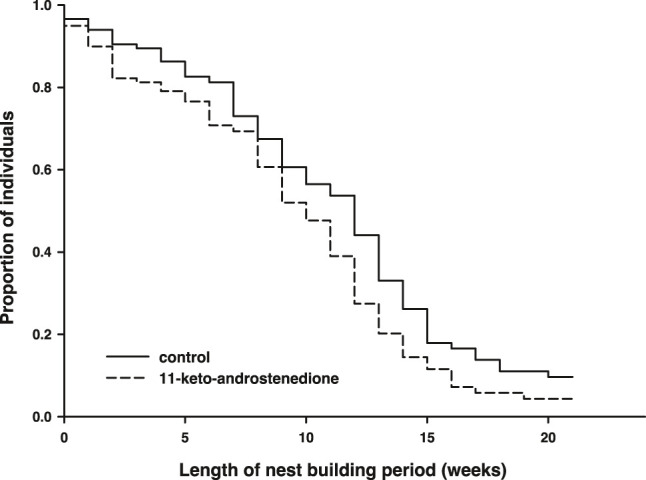
Period (in weeks) during which reproductive activities could be maintained was reduced in individuals in which 11-ketotestosterone was elevated (dashed line) compared to controls (closed line).

**FIGURE 5:**
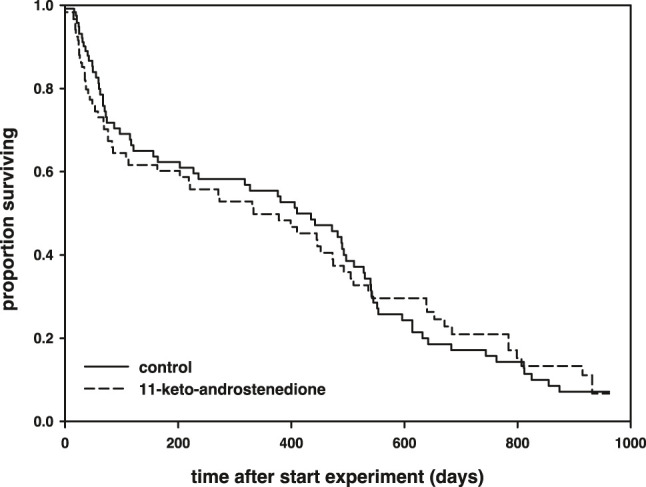
Survival (in days after the start of the experiment) plotted across the whole follow-up period, separated for 11-keto-androstenedione-treated individuals (dashed) and controls (solid). The breeding season from which data on coloration and nest building are presented lasted for 160 days of long photoperiod after the start of the experiment.

11-Keto-androstenedione treatment accelerated reproductive senescence. The period that individuals maintained their breeding activities (week of last complete nest minus week of first complete nest) was shorter after androgen treatment (logHR = 0.35 ± 0.16, *p* = 0.034, Figure 4), whereas the interaction with the main effect of vitamin E treatment was not significant (*p* > 0.22) and therefore removed from the model. This effect did not arise from 11-keto-androstenedione-treated animals starting with nest building sooner (rank test, *W* = 6,985.5, *p* = 0.45). A small part of this effect can be attributed to lower survival in the 11-keto-androstenedione treated fish. When analyzed across all data available for this study, there is a small decrease in survival that is far from significant (logHR = 0.05 ± 0.17, *p* = 0.75; Figure 5). Yet, when we analyzed data of the breeding season only, or up to the point of which we are sure 11-ketotestosterone is elevated (the last point at which animals were sacrificed for this purpose) the reduced survival in the 11-keto-androstenedione group these effects were stronger, but remained non-significant (logHR = 0.24 ± 0.25, *p* = 0.33, logHR = 0.53 ± 0.38, *p* = 0.16, respectively). No effects of vitamin E were detected in any of these models (*p* > 0.24).

### Body Composition

Individual mass at sacrificing covaried positively with all four organ masses (*p* < 0.0002) and was therefore included in the models testing for treatment effects. 11-Keto-androstenedione treatment and vitamin E treatment did not interact for any of the organ masses (*p* > 0.14) and the week at which the individual was killed did not contribute significantly either (*p* > 0.08). Therefore, we tested the effect of 11-keto-androstenedione and vitamin E treatment separately on the masses of the testes, liver, kidney, and spleen, and also on mass at sacrificing with mass at the start of treatment included as covariate (Table 1). No effects of 11-keto-androstenedione treatment were detected. Vitamin E treatment reduced mass-specific liver and spleen mass, and also mass change from mass at sacrificing (Table 1). Note that most of these effects are likely driven by mass loss compared to mass at injection (mass at injection was balanced but slightly higher in vitamin E treated fish, estimate: 0.013 ± 0.11, *p* = 0.23). When mass at sacrificing was removed from the models, all associations with vitamin E and organ masses were reduced to non-significant trends, *p* > 0.07.

**TABLE 1:** Effects of treatment with 11-keto-androstenedione (11 kA) or vitamin E on organ mass (including mass at sacrificing as covariate) and mass at sacrificing and mass change (including mass at injection as covariate).

	Treatment	
	11 kA	Vitamin E
Testes	−0.0017 (0.0012)	−0.0010 (0.0012)
*p*	0.18	0.41
Liver	−0.0033 (0.0056)	−0.014 (0.0054)
*p*	0.56	0.011
Kidney	−0.0049 (0.0036)	−0.0044 (0.0036)
*p*	0.18	0.23
Spleen	−0.00037 (0.00097)	−0.0017 (0.00095)
*p*	0.71	0.07
Mass at sacrifice	−0.0053 (0.10)	−0.052 (0.10)
*p*	0.96	0.61
Mass change	−0.0072 (0.044)	−0.094 (0.044)
*p*	0.87	0.036

Estimates are given of the treatment effects with their standard errors, within parentheses. Sample size is between 76 and 79 individual fish due to missing data. Note that this sample size is higher than the sample sizes that we could use for the 11-ketotestosterone analyses, because of failures in collecting or processing blood.

### Coloration

**FIGURE 6:**
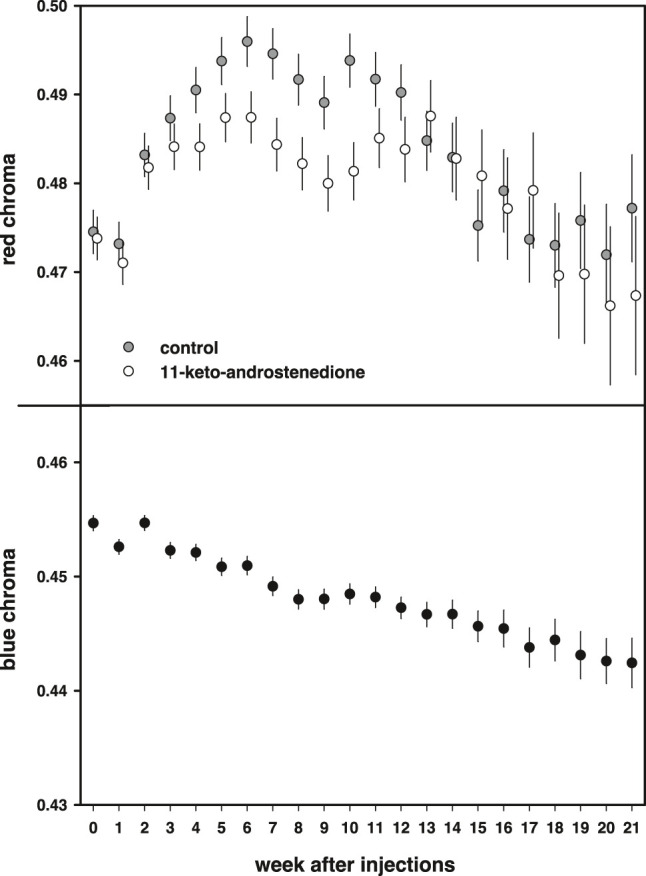
Breeding coloration plotted against the weeks after injections. Red chroma of the belly is plotted in the top panel and first increases during the breeding season to subsequently decline after an optimum. 11-Keto-androstenedione-treated animals have slightly less concentrated coloration of their bellies before and after the period of the optimum. The bottom panel shows blue iris coloration, which steadily declines during the breeding season irrespective of treatment.

Red coloration increased during the first part of the breeding season and declined at the end [week: *F*(21,1663) = 14.8, *p* < 0.0001]. This increase was lower in the androgen-treated fish, resulting in lower red coloration during the middle of the breeding season, but this effect did not reach statistical significance [11 kA × week: *F*(21,1663) = 1.46, *p* = 0.08; Figure 6]. In the models, we also included mass at the start of the experiment, which covaried positively with the intensity of the red breeding coloration [estimate: 0.010 ± 0.003, *F*(1,220.2) = 10.90, *p* = 0.001]. In these models, we did not detect any effects or interactions with vitamin E treatment (*p* > 0.70).

Blue coloration decreased during the breeding season [*F*(21,1536) = 13.8, *p* < 0.0001; Figure 6] but no effects or interactions with either 11-keto-androstenedione or vitamin E treatment were detected (*p* > 0.72) and no relationship with mass was detected [estimate: 0.0017 ± 0.001, *F*(1,220.8) = 2.64, *p* = 0.11].

### Nest Building Behavior

**FIGURE 7:**
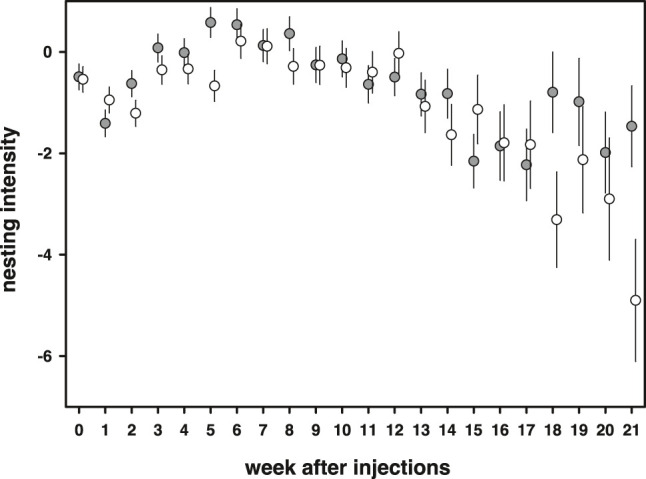
Nesting intensity [amount of days extra material was added minus the time needed to complete a nest (days)] first increased during the breeding season and then declined. 11-Keto-androstenedione (open dots)-treated individuals showed in general reduced nesting intensity during the start of the breeding season.

Nesting intensity, the amount of times extra material was added minus the time needed to complete a nest, increased at the start of the breeding season and gradually declined towards the end of the season [week: *F*(21,1609) = 6.18, *p* < 0.0001, Figure 7]. 11-Keto-androstenedione-treated individuals showed reduced nesting intensity at the beginning and end of the breeding season [week × 11 kA: *F*(21,1609) = 1.60, *p* = 0.042; Figure 7]. No interactions with vitamin E treatment were detected (*p* > 0.47) and mass was positively related to nesting intensity [estimate = 0.94 ± 0.25, *F*(1,203.8) = 13.6, *p* = 0.0003].

### Relationships Between the Intensity of Breeding Coloration With Reproductive Senescence and Lifespan

**FIGURE 8:**
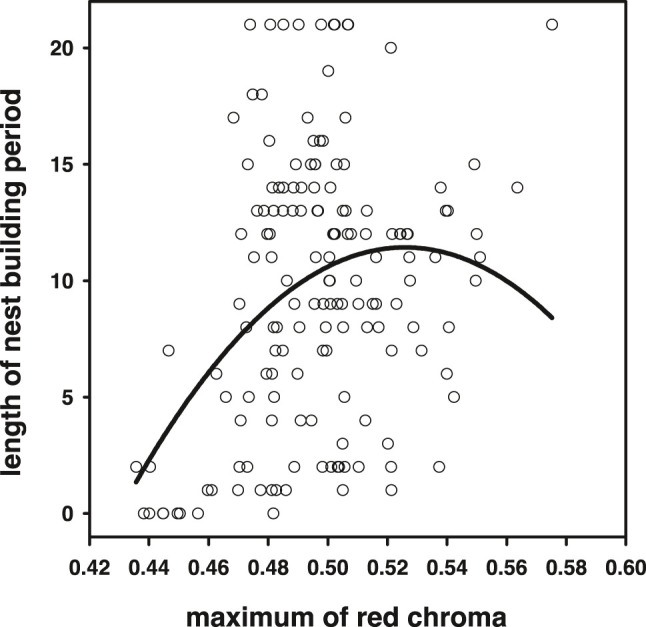
Higher maximum red chroma achieved during the breeding season signaled higher reproductive capacity. However, the significance of the quadratic term suggests that in the reddest individuals, this relationship levels off or may even become negative.

Average and maximum chroma (which correlated strongly, *r* = 0.94, *N* = 155, *p* < 0.0001) achieved during the breeding season correlated positively with the time individuals could maintain their nesting activity (average: *r* = 0.17, *p* = 0.036; maximum *r* = 0.29, *p* = 0.0002, *N* = 155; Figure 8). For maximum blue coloration, we also detected a trend of a positive relationship (*r* = 0.15, *p* = 0.06, *N* = 155, but not for average *r* = −0.12, *p* = 0.12, *N* = 155). Note that from these analyses, we excluded individuals that were sacrificed for the 11-ketotestosterone analyses, because these animals are censored with respect to the averaged and maximum chroma they could have achieved. Because quadratic relationships with sexual signal expression and longevity have been reported previously, we also tested for quadratic effects and detected a quadratic effect in the relationship between maximum red chroma and breeding period [*F*(1,152) = 7.73, *p* = 0.006; Figure 8, quadratic relationships with max blue chroma were not significant *p* > 0.9]. Note that this quadratic relationship with breeding period (and also lifespan below) and maximum chroma cannot be attributable to regression to the mean. The maximum is predicted to increase with the number of sampling points, related to breeding period (and lifespan) in our setup, and in this respect, the linear slope of maximum chroma may be biased upward, rather than downward.

Lifespan was also positively related to average [logHR: −3.29 ± 4.06, *χ*(1)^2^ = 0.67, *p* = 0.42] and maximum red chroma [logHR: −9.20 ± 4.00, *χ*(1)^2^ = 5.50, *p* = 0.022]. For maximum red chroma, we also detected a quadratic relationship (logHR estimates: linear: −290 ± 90.7, *p* = 0.001; quadratic 279 ± 89.8, *p* < 0.002). Higher maximum blue chroma tended to be associated with lower survival (logHR: 16.64 ± 10.90, *p* = 0.13). When maximum blue chroma was added to the quadratic maximum red chroma model, both the linear term and the quadratic term of red chroma remained significant (*p* < 0.008), and maximum blue chroma was significantly positively related to mortality hazard (logHR: 27.7 ± 12.3, *p* = 0.024). Note that maximum red chroma and maximum blue chroma only loosely correlate (*r =* 0.35, *p* < 0.001) (Rush et al., 2003); multicollinearity biasing the model estimates is therefore unlikely.

## Discussion

### Current and Future Reproduction

11-Ketotestosterone elevation decreased our proxy for future reproductive success, the length that reproductive activities are maintained. This suggests that androgens are involved in the regulation of the trade-off between current and future reproduction. However, we do not find any evidence for positive effects on our proxies for current reproduction after 11-ketotestosterone elevation. On the contrary, if anything, red breeding coloration and nesting vigor are lowered in the 11-ketotestosterone elevated animals. Negative consequences of our treatment because of high pharmacological dosing are unlikely because our treatment resulted in only a mild increase in 11-ketotestosterone. Therefore, investment induced by 11-ketotestosterone in other aspects of physiology that we did not measure may have generated the costs that resulted in the reduction in future reproductive success we observed. These costs could be related to various aspects of reproduction such as sperm production, spiggin production, and sexual behavior. It is probably unlikely that these costs involve direct increased metabolic demand, because mass and body composition were left unaffected by 11-ketotestosterone elevation. Alternatively, there might be direct costs of higher levels of 11-ketotestosterone imposed by unknown physiological constraints, thereby reducing future reproduction.

### Androgens, Vitamin E, and Carotenoid-Dependent Coloration

The hypothesized involvement of androgens in the trade-offs concerning carotenoid-dependent coloration (Alonso-Alvarez et al., 2007; Peters, 2007) is not supported by our findings. Elevation of 11-ketotestosterone did not increase red breeding coloration, but rather decreased it. In addition, variation in 11-ketotestosterone levels was not related to breeding coloration, in line with an earlier smaller study (Wright et al., 2016). Furthermore, another study on stickleback red breeding coloration and 11-ketotestosterone (Kurtz et al., 2007) found that if individuals had spent 4 (higher 11-ketotestosterone level) compared to 6 weeks (lower 11-ketotestosterone levels) in breeding conditions prior to measurement of circulating 11-ketotestosterone levels, a correlation with breeding coloration was not apparent. Our results and previous ones might thus suggest that variation in 11-ketotestosterone levels in full breeding condition does not determine investment in breeding coloration but may still regulate a suite of other behaviors or aspects of physiology related to current reproduction.

Our results also do not support the mechanistic explanation for the hypothesized trade-off between oxidative stress and carotenoid allocation to sexual coloration. Vitamin E treatment did not increase coloration. This is contrary to an earlier report in stickleback showing that breeding coloration increased under a diet of a combination of vitamin C and E (Pike et al., 2007a). Similarly, the reduction in time that 11-keto-androstenedione-treated fish could maintain their reproductive activities is unlikely to be attributable to oxidative stress costs, because we detected no interactions with the vitamin E treatment. These conclusions all assume that our methodology of injecting vitamin E resulted in elevated levels of vitamin E in our fish. No pellets were lost by fish and were observed in all fish that were sacrificed and died a natural death. We must assume that vitamin E was therefore available to the fish for uptake. However, we prioritized analyzing the plasma samples for 11-ketotestosterone analyses as they were too small to also measure vitamin E. Treatment with vitamin E led to slightly lower body and organ mass, which could indicate a loss rather than a gain in body condition. Our conclusions are therefore dependent on the only partially supported assumption that vitamin E injection led to increased bioavailable vitamin E.

### Senescence of Nuptial Coloration and Associations With Survival

Variation in red breeding coloration was positively related to longevity and the time nesting activities could be maintained. This is in concordance with an earlier study on sticklebacks of a smaller sample size (*N* = 32) that investigated the relationship between redness and longevity (Pike et al., 2007b). Sexual ornament expression is in general found to be positively related to survival (Jennions et al., 2001), yet examples for carotenoid-dependent coloration are relatively scarce and dominated by studies on birds (Hill, 1991; Hõrak et al., 2001; Figuerola and Carlos Senar, 2007; Simons et al., 2012d; Simons et al., 2016; Cantarero et al., 2019). Interestingly, we detected a quadratic relationship of redness with survival and the length that breeding can be maintained, suggesting that, at a certain point, carotenoid-dependent breeding coloration does not signal quality but may be related to reduced survival and breeding capacity. It is possible that this is a common pattern for carotenoid-dependent signals or even sexual signals in general. Serins (*Serinus serinus*) with intermediate carotenoid-derived brightness have higher survival (Figuerola and Carlos Senar, 2007), and in the zebra finch (*Taeniopygia guttata*), we detected a similar quadratic relationship of their carotenoid-based bill coloration and longevity, when controlling for terminal declines (Simons et al., 2012d; Simons et al., 2016). Together, these findings indicate that the reddest mates potentially overinvest in their ornament to attract females. Choice for these males (Milinski and Bakker, 1990; Künzler and Bakker, 2001; Pike et al., 2007b) might still provide the direct benefits of producing sexy sons, or high signaling males might signal different aspects of quality, perhaps related more to current rather than future reproduction. For example, redness of the stickleback belly is related to functional fertility (Pike et al., 2010) and unexpectedly higher somatic and germline damage was detected in males with the most red signals (Kim and Velando, 2020).

Compared to the carotenoid-based red belly of the stickleback, their iridescent blue iris has been studied less, but has been suggested to be a decisive feature during female mate choice (Rush et al., 2003; Flamarique et al., 2013). Males may maintain a red belly for the purpose of enhancing blue iris contrast (Rush et al., 2003; Flamarique et al., 2013). Surprisingly we find that chroma of the blue iris reduces over the breeding season and that red breeding coloration is not at its maximum when the blue iris is. There is thus a mismatch between these two signals that does not result in the highest possible contrast, which would be perceived as most attractive to females. Moreover, the maximum intensity of the blue iris was negatively related to survival, which can suggest that there are costs to maintain iris color. The iridescent blue iris of the stickleback is formed by endogenously produced pigments (Frischknecht, 1993). The associated costs or detailed information on its physiology is currently lacking and warrant future study.

## Conclusion

11-Ketotestosterone elevation reduced future reproductive success. Although we did not detect benefits to current reproduction, it is plausible that 11-ketotestosterone regulates aspects of the trade-off between current and future reproduction. It remains to be determined what these aspects are. We did not detect any evidence for the proposed regulatory role of androgens in the carotenoid trade-off between somatic maintenance and sexual signaling. We did find, however, that carotenoid-dependent coloration signals reproductive capacity and longevity. In the reddest individuals, however, this relationship is diminished and even turns negative. These quadratic relationships between sexual signal expression and aspects of quality (Candolin, 1999; Simons et al., 2016), not unique to the stickleback, have important consequences for how we view sexual selection on ornamentation in general.

## Data Availability

The raw data supporting the conclusion of this article will be made available by the authors, without undue reservation.

## References

[B1] Abrams P. A. (1991). The Fitness Costs of Senescence: The Evolutionary Importance of Events in Early Adult Life. Evol. Ecol. 5, 343–360. 10.1007/bf02214152

[B2] Alonso-Alvarez C. Bertrand S. Faivre B. Chastel O. Sorci G. (2007). Testosterone and Oxidative Stress: The Oxidation Handicap Hypothesis. Proc. R. Soc. B. 274, 819–825. 10.1098/rspb.2006.3764 PMC2093982 17251089

[B3] Alonso-Alvarez C. Cantarero A. Romero-Haro A. Á. Chastel O. Pérez-Rodríguez L. (2020). Life-Long Testosterone and Antiandrogen Treatments Affect the Survival and Reproduction of Captive Male Red-Legged Partridges (*Alectoris rufa*). Behav. Ecol. Sociobiol. 74, 98. 10.1007/s00265-020-02878-1

[B4] Augustine Jacqueline, K. Millspaugh Joshua, J. Sandercock Brett K. (2011). “Chapter Fourteen. Testosterone Mediates Mating Success in Greater Prairie-Chickens,” in Ecology, Conservation, and Management of Grouse: Published for the Cooper Ornithological Society. Editors Brett K. S. Kathy Martin Gernot Segelbacher. (Berkeley: University of California Press), 195–208. 10.1525/9780520950573-016

[B5] Barber I. (2001). Nests as Ornaments: Revealing Construction by Male Sticklebacks. Behav. Ecol. 12, 390–396. 10.1093/beheco/12.4.390

[B6] Bell A. M. (2020). Individual Variation and the Challenge Hypothesis. Horm. Behav. 123, 104549. 10.1016/j.yhbeh.2019.06.013 31247185 PMC6980443

[B7] Blas J. Perez-Rodriguez L. Bortolotti G. R. Vinuela J. Marchant T. A. (2006). Testosterone Increases Bioavailability of Carotenoids: Insights into the Honesty of Sexual Signaling. Proc. Natl. Acad. Sci. 103, 18633–18637. 10.1073/pnas.0609189103 17121984 PMC1660487

[B8] Boonekamp J. J. Ros A. H. F. Verhulst S. (2008). Immune Activation Suppresses Plasma Testosterone Level: A Meta-Analysis. Biol. Lett. 4, 741–744. 10.1098/rsbl.2008.0347 18700197 PMC2614147

[B9] Borg B. (1994). Androgens in Teleost Fishes. Comp. Biochem. Physiol. C: Pharmacol. Toxicol. Endocrinol. 109, 219–245. 10.1016/0742-8413(94)00063-g

[B10] Borg B. Antonopoulou E. Andersson E. Carlberg T. Mayer I. (1993). Effectiveness of Several Androgens in Stimulating Kidney Hypertrophy, a Secondary Sexual Character, in Castrated Male Three-Spined Sticklebacks, *Gasterosteus aculeatus* . Can. J. Zool. 71, 2327–2329. 10.1139/z93-326

[B11] Borg B. Mayer I. (1995). Androgens and Behaviour in the Three-Spined Stickleback. Behaviour 132, 1025–1035. 10.1163/156853995x00432

[B12] Briga M. Verhulst S. (2015). What Can Long-Lived Mutants Tell Us about Mechanisms Causing Aging and Lifespan Variation in Natural Environments? Exp. Gerontol. 71, 21–26. 10.1016/j.exger.2015.09.002 26362217

[B13] Buttemer W. A. Warne S. Bech C. Astheimer L. B. (2008). Testosterone Effects on Avian Basal Metabolic Rate and Aerobic Performance: Facts and Artefacts. Comp. Biochem. Physiol. A Mol. Integr. Physiol. 150, 204–210. 10.1016/j.cbpa.2006.06.047 17107819

[B14] Candolin U. (1999). The Relationship between Signal Quality and Physical Condition: Is Sexual Signalling Honest in the Three-Spined Stickleback? Anim. Behav. 58, 1261–1267. 10.1006/anbe.1999.1259 10600148

[B15] Cantarero A. Pérez-Rodríguez L. Romero-Haro A. Á. Chastel O. Alonso-Alvarez C. (2019). Carotenoid-based Coloration Predicts Both Longevity and Lifetime Fecundity in Male Birds, but Testosterone Disrupts Signal Reliability. PLoS One 14, e0221436. 10.1371/journal.pone.0221436 31442265 PMC6707625

[B16] Casagrande S. Costantini D. Dell’Omo G. Tagliavini J. Groothuis T. G. G. (2012). Differential Effects of Testosterone Metabolites Oestradiol and Dihydrotestosterone on Oxidative Stress and Carotenoid-Dependent Colour Expression in a Bird. Behav. Ecol. Sociobiol. 66, 1319–1331. 10.1007/s00265-012-1387-3

[B17] Costantini D. Møller A. P. (2009). Does Immune Response Cause Oxidative Stress in Birds? A Meta-Analysis. Comp. Biochem. Physiol. A Mol. Integr. Physiol. 153, 339–344. 10.1016/j.cbpa.2009.03.010 19303455

[B18] Daan S. Dijkstra C. Tinbergen J. M. (1990). Family Planning in the Kestrel (*Falco tinnunculus*): The Ultimate Control of Covariation of Laying Date and Clutch Size. Behaviour 114, 83–116. 10.1163/156853990x00077

[B19] Dijkstra C. Bult A. Bijlsma S. Daan S. Meijer T. Zijlstra M. (1990). Brood Size Manipulations in the Kestrel (*Falco tinnunculus*): Effects on Offspring and Parent Survival. J. Anim. Ecol. 59, 269. 10.2307/5172

[B20] Dufty A. M. (1989). Testosterone and Survival: A Cost of Aggressiveness. Horm. Behav. 23, 185–193. 10.1016/0018-506x(89)90059-7 2744737

[B21] Emlen D. J. Warren I. A. Johns A. Dworkin I. Lavine L. C. (2012). A Mechanism of Extreme Growth and Reliable Signaling in Sexually Selected Ornaments and Weapons. Science 337, 860–864. 10.1126/science.1224286 22837386

[B22] Figuerola J. Carlos Senar J. (2007). Serins with Intermediate Brightness Have a Higher Survival in the Wild. Oikos 116, 636–641. 10.1111/j.0030-1299.2007.14719.x

[B23] Flamarique I. N. Bergstrom C. Cheng C. L. Reimchen T. E. (2013). Role of the Iridescent Eye in Stickleback Female Mate Choice. J. Exp. Biol. 216, 2806–2812. 10.1242/jeb.084889 23580716

[B24] Flatt T. (2011). Survival Costs of Reproduction in Drosophila. Exp. Gerontol. 46, 369–375. 10.1016/j.exger.2010.10.008 20970491

[B25] Folstad I. Karter A. J. (1992). Parasites, Bright Males, and the Immunocompetence Handicap. The Am. Naturalist 139, 603–622. 10.1086/285346

[B26] Frischknecht M. (1993). The Breeding Colouration of Male Three-Spined Sticklebacks (*Gasterosteus aculeatus*) as an Indicator of Energy Investment in Vigour. Evol. Ecol. 7, 439–450. 10.1007/bf01237640

[B27] Garratt M. Try H. Smiley K. O. Grattan D. R. Brooks R. C. (2020). Mating in the Absence of Fertilization Promotes a Growth-Reproduction versus Lifespan Trade-Off in Female Mice. Proc. Natl. Acad. Sci. U.S.A. 117, 15748–15754. 10.1073/pnas.2003159117 32571943 PMC7355008

[B28] Giraudeau M. Sweazea K. Butler M. W. McGraw K. J. (2013). Effects of Carotenoid and Vitamin E Supplementation on Oxidative Stress and Plumage Coloration in House Finches (*Haemorhous mexicanus*). Comp. Biochem. Physiol. A Mol. Integr. Physiol. 166, 406–413. 10.1016/j.cbpa.2013.07.014 23872319

[B29] Grafen A. (1990). Biological Signals as Handicaps. J. Theor. Biol. 144, 517–546. 10.1016/s0022-5193(05)80088-8 2402153

[B30] Hau M. (2007). Regulation of Male Traits by Testosterone: Implications for the Evolution of Vertebrate Life Histories. BioEssays 29, 133–144. 10.1002/bies.20524 17226801

[B31] Hellqvist A. Mayer I. Borg B. (2002). Effects of Hemi-Castration on Plasma Steroid Levels in Two Teleost Fishes; the Three-Spined Stickleback, *Gasterosteus aculeatus*, and the Atlantic salmon, *Salmo salar* . Fish. Physiol. Biochem. 26, 107–110. 10.1023/A:1025435625551

[B32] Hill G. E. (1991). Plumage Coloration Is a Sexually Selected Indicator of Male Quality. Nature 350, 337–339. 10.1038/350337a0

[B33] Höglund J. Sheldon B. C. Hoglund J. (1998). The Cost of Reproduction and Sexual Selection. Oikos 83, 478, 10.2307/3546675

[B34] Holtmann B. Lagisz M. Nakagawa S. (2017). Metabolic Rates, and Not Hormone Levels, are a Likely Mediator of Between‐individual Differences in Behaviour: A Meta‐Analysis. Funct. Ecol. 31, 685–696. 10.1111/1365-2435.12779

[B35] Hõrak P. Ots I. Vellau H. Spottiswoode C. Pape Møller A. (2001). Carotenoid-based Plumage Coloration Reflects Hemoparasite Infection and Local Survival in Breeding Great Tits. Oecologia 126, 166–173. 10.1007/s004420000513 28547614

[B36] Jakobsson S. Borg B. Haux C. Hyllner S. J. (1999). An 11-ketotestosterone Induced Kidney-Secreted Protein: the Nest Building Glue from Male Three-Spined Stickleback, *Gasterosteus aculeatus* . Fish. Physiol. Biochem. 20, 79–85. 10.1023/a:1007776016610

[B37] Jakobsson S. Mayer I. Schulz R. W. Blankenstein M. A. Borg B. (1996). Specific Binding of 11-ketotestosterone in an Androgen Target Organ, the Kidney of the Male Three-Spined Stickleback, *Gasterosteus aculeatus* . Fish. Physiol. Biochem. 15, 459–467. 10.1007/bf01874920 24194355

[B38] Jennions M. D. Moller A. P. Petrie M. (2001). Sexually Selected Traits and Adult Survival: a Meta-Analysis. Q. Rev. Biol. 76, 3–36. 10.1086/393743 11291569

[B39] Karu U. Saks L. Hõrak P. (2008). Carotenoid-based Plumage Coloration Is Not Affected by Vitamin E Supplementation in Male Greenfinches. Ecol. Res. 23, 931–935. 10.1007/s11284-007-0457-x

[B40] Khalil S. Welklin J. F. McGraw K. J. Boersma J. Schwabl H. Webster M. S. (2020). Testosterone Regulates CYP2J19-Linked Carotenoid Signal Expression in Male Red-Backed Fairywrens (*Malurus melanocephalus*). Proc. Biol. Sci. 287, 20201687. 10.1098/rspb.2020.1687 32933448 PMC7542802

[B41] Kim S. Y. Metcalfe N. B. Velando A. (2016). A Benign Juvenile Environment Reduces the Strength of Antagonistic Pleiotropy and Genetic Variation in the Rate of Senescence. J. Anim. Ecol. 85, 705–714. 10.1111/1365-2656.12468 26559495 PMC4991295

[B42] Kim S. Y. Velando A. (2020). Attractive Male Sticklebacks Carry More Oxidative DNA Damage in the Soma and Germline. J. Evol. Biol. 33, 121–126. 10.1111/jeb.13552 31610052

[B43] Kirkwood T. B. L. (2002). Evolution of Ageing. Mech. Ageing Dev. 123, 737–745. 10.1016/s0047-6374(01)00419-5 11869731

[B44] Koch R. E. Kavazis A. N. Hasselquist D. Hood W. R. Zhang Y. Toomey M. B. (2018). No Evidence that Carotenoid Pigments Boost Either Immune or Antioxidant Defenses in a Songbird. Nat. Commun. 9, 491–497. 10.1038/s41467-018-02974-x 29403051 PMC5799171

[B45] Koch R. E. Hill G. E. (2018). Do Carotenoid‐Based Ornaments Entail Resource Trade‐offs? an Evaluation of Theory and Data. Funct. Ecol. 32, 1908–1920. 10.1111/1365-2435.13122

[B46] Kokko H. (1999). Competition for Early Arrival in Migratory Birds. J. Anim. Ecol. 68, 940–950. 10.1046/j.1365-2656.1999.00343.x

[B47] Kotiaho J. S. (2001). Costs of Sexual Traits: A Mismatch Between Theoretical Considerations and Empirical Evidence. Biol. Rev. 76, 365–376. 10.1017/s1464793101005711 11569789

[B48] Künzler R. Bakker T. C. M. (2001). Female Preferences for Single and Combined Traits in Computer Animated Stickleback Males. Behav. Ecol. 12, 681–685. 10.1093/beheco/12.6.681

[B49] Kurtz J. Kalbe M. Langefors Å Mayer I. Milinski M. Hasselquist D. (2007). An Experimental Test of the Immunocompetence Handicap Hypothesis in a Teleost Fish: 11‐Ketotestosterone Suppresses Innate Immunity in Three‐Spined Sticklebacks. Am. Nat. 170, 509–519. 10.1086/521316 17891730

[B50] Lemaître J. F. Berger V. Bonenfant C. Douhard M. Gamelon M. Plard F. (2015). Early-late Life Trade-Offs and the Evolution of Ageing in the Wild. Proc. R. Soc. B Biol. Sci. 282, 20150209. 10.1098/rspb.2015.0209 PMC4426628 25833848

[B51] Lind M. I. Carlsson H. Duxbury E. M. L. Ivimey-Cook E. Maklakov A. A. (2021). Cost-free Lifespan Extension via Optimization of Gene Expression in Adulthood Aligns with the Developmental Theory of Ageing. Proc. R. Soc. B Biol. Sci. 288, 20201728. 10.1098/rspb.2020.1728 PMC7893226 33529563

[B52] Maklakov A. A. Chapman T. (2019). Evolution of Ageing as a Tangle of Trade-Offs: Energy versus Function. Proc. Biol. Sci. 286, 20191604. 10.1098/rspb.2019.1604 31530150 PMC6784717

[B53] Marler C. A. Moore M. C. (1991). Supplementary Feeding Compensates for Testosterone-Induced Costs of Aggression in Male Mountain Spiny Lizards, Sceloporus Jarrovi. Anim. Behav. 42, 209–219. 10.1016/s0003-3472(05)80552-4

[B54] McCracken A. W. Adams G. Hartshorne L. Tatar M. Simons M. J. P. (2020). The Hidden Costs of Dietary Restriction: Implications for its Evolutionary and Mechanistic Origins. Sci. Adv. 6, eaay3047. 10.1126/sciadv.aay3047 32128403 PMC7034997

[B55] McCracken A. W. Buckle E. Simons M. J. P. (2020). The Relationship between Longevity and Diet is Genotype Dependent and Sensitive to Desiccation in *Drosophila melanogaster* . J. Exp. Biol. 223, jeb230185. 10.1242/jeb.230185 33109715 PMC7725603

[B56] Milinski M. Bakker T. C. M. (1990). Female Sticklebacks Use Male Coloration in Mate Choice and Hence Avoid Parasitized Males. Nature 344, 330–333. 10.1038/344330a0

[B57] Moss R. Parr R. Lambin X. (1994). Effects of Testosterone on Breeding Density, Breeding success and Survival of Red Grouse. Proc. R. Soc. Lond. B 258, 175–180. 10.1098/rspb.1994.0159

[B58] Mougeot F. Irvine J. R. Seivwright L. Redpath S. M. Piertney S. (2004). Testosterone, Immunocompetence, and Honest Sexual Signaling in Male Red Grouse. Behav. Ecol. 15, 930–937. 10.1093/beheco/arh087

[B59] Mougeot F. Piertney S. B. Leckie F. Evans S. Moss R. Redpath S. M. (2005). Experimentally Increased Aggressiveness Reduces Population Kin Structure and Subsequent Recruitment in Red Grouse Lagopus Lagopus Scoticus. J. Anim. Ecol. 74, 488–497. 10.1111/j.1365-2656.2005.00947.x

[B60] Newhouse D. J. Vernasco B. J. (2020). Developing a Transcriptomic Framework for Testing Testosterone-Mediated Handicap Hypotheses. Gen. Comp. Endocrinol. 298, 113577. 10.1016/j.ygcen.2020.113577 32739436

[B61] Páll M. K. Mayer I. Borg B. (2002). Androgen and Behavior in the Male Three-Spined Stickleback, *Gasterosteus aculeatus* I. - Changes in 11-ketotestosterone Levels during the Nesting Cycle. Horm. Behav. 41, 377–383. 10.1006/hbeh.2002.1777 12018933

[B62] Pérez C. Lores M. Velando A. (2008). Availability of Nonpigmentary Antioxidant Affects Red Coloration in Gulls. Behav. Ecol. 19, 967–973. 10.1093/beheco/arn053

[B63] Perez-Rodriguez L. Mougeot F. Alonso-Alvarez C. Blas J. Viñuela J. Bortolotti G. R. (2008). Cell-mediated Immune Activation Rapidly Decreases Plasma Carotenoids but Does Not Affect Oxidative Stress in Red-Legged Partridges (*Alectoris rufa*). J. Exp. Biol. 211, 2155–2161. 10.1242/jeb.017178 18552305

[B64] Peters A. (2007). Testosterone and Carotenoids: An Integrated View of Trade-Offs between Immunity and Sexual Signalling. BioEssays 29, 427–430. 10.1002/bies.20563 17450573

[B65] Pike T. W. Blount J. D. Bjerkeng B. Lindström J. Metcalfe N. B. (2007). Carotenoids, Oxidative Stress and Female Mating Preference for Longer Lived Males. Proc. R. Soc. B. 274, 1591–1596. 10.1098/rspb.2007.0317 PMC2169282 17439854

[B66] Pike T. W. Blount J. D. Lindström J. Metcalfe N. B. (2007). Availability of Non-Carotenoid Antioxidants Affects the Expression of a Carotenoid-Based Sexual Ornament. Biol. Lett. 3, 353–356. 10.1098/rsbl.2007.0072 17472903 PMC2390655

[B67] Pike T. W. Blount J. D. Lindström J. Metcalfe N. B. (2010). Dietary Carotenoid Availability, Sexual Signalling and Functional Fertility in Sticklebacks. Biol. Lett. 6, 191–193. 10.1098/rsbl.2009.0815 19923137 PMC2865065

[B68] Pike T. W. (2011). Using Digital Cameras to Investigate Animal Colouration: Estimating Sensor Sensitivity Functions. Behav. Ecol. Sociobiol. 65, 849–858. 10.1007/s00265-010-1097-7

[B69] Redpath S. M. Mougeot F. Leckie F. M. Evans S. A. (2006). The Effects of Autumn Testosterone on Survival and Productivity in Red Grouse, Lagopus Lagopus Scoticus. Anim. Behav. 71, 1297–1305. 10.1016/j.anbehav.2005.08.012

[B70] Reed W. L. Clark M. E. Parker P. G. Raouf S. A. Arguedas N. Monk D. S. (2006). Physiological Effects on Demography: A Long‐Term Experimental Study of Testosterone's Effects on Fitness. Am. Nat. 167, 667–683. 10.1086/503054 16671011

[B71] Regan J. C. Froy H. Walling C. A. Moatt J. P. Nussey D. H. (2019). Dietary Restriction and Insulin‐like Signalling Pathways as Adaptive Plasticity: A Synthesis and Re‐evaluation. Funct. Ecol. 34, 107–128. 10.1111/1365-2435.13418

[B72] Reznick D. Nunney L. Tessier A. (2000). Big Houses, Big Cars, Superfleas and the Costs of Reproduction. Trends Ecol. Evol. 15, 421–425. 10.1016/s0169-5347(00)01941-8 10998520

[B73] Roberts M. L. Buchanan K. L. Evans M. R. (2004). Testing the Immunocompetence Handicap Hypothesis: A Review of the Evidence. Anim. Behav. 68, 227–239. 10.1016/j.anbehav.2004.05.001

[B74] Rush V. Abney M. McKinnon J. Sargent R. C. (2003). Reflectance Spectra from Free-Swimming Sticklebacks (Gasterosteus): Social Context and Eye-Jaw Contrast. Behaviour 140, 1003–1019. 10.1163/156853903322589614

[B75] Rushbrook B. J. Dingemanse N. J. Barber I. (2008). Repeatability in Nest Construction by Male Three-Spined Sticklebacks. Anim. Behav. 75, 547–553. 10.1016/j.anbehav.2007.06.011

[B76] Rutten A. L. Oosterbeek K. Ens B. J. Verhulst S. (2006). Optimal Foraging on Perilous Prey: Risk of Bill Damage Reduces Optimal Prey Size in Oystercatchers. Behav. Ecol. 17, 297–302. 10.1093/beheco/arj029

[B77] Salvador A. Veiga J. P. Martin J. Lopez P. Abelenda M. Puertac M. (1996). The Cost of Producing a Sexual Signal: Testosterone Increases the Susceptibility of Male Lizards to Ectoparasitic Infestation. Behav. Ecol. 7, 145–150. 10.1093/beheco/7.2.145

[B78] Santos E. S. A. Nakagawa S. (2012). The Costs of Parental Care: A Meta-Analysis of the Trade-Off between Parental Effort and Survival in Birds. J. Evol. Biol. 25, 1911–1917. 10.1111/j.1420-9101.2012.02569.x 22830387

[B79] Schantz T. v. Bensch S. Grahn M. Hasselquist D. Wittzell H. (1999). Good Genes, Oxidative Stress and Condition-Dependent Sexual Signals. Proc. R. Soc. Lond. B 266, 1–12. 10.1098/rspb.1999.0597 PMC1689644 10081154

[B80] Schielzeth H. Forstmeier W. (2009). Conclusions Beyond Support: Overconfident Estimates in Mixed Models. Behav. Ecol. 20, 416–420. 10.1093/beheco/arn145 19461866 PMC2657178

[B81] Sebire M. Katsiadaki I. Scott A. P. (2007). Non-Invasive Measurement of 11-ketotestosterone, Cortisol and Androstenedione in Male Three-Spined Stickleback (*Gasterosteus aculeatus*). Gen. Comp. Endocrinol. 152, 30–38. 10.1016/j.ygcen.2007.02.009 17412342

[B82] Sheldon B. C. Verhulst S. (1996). Ecological Immunology: Costly Parasite Defences and Trade-Offs in Evolutionary Ecology. Trends Ecol. Evol. 11, 317–321. 10.1016/0169-5347(96)10039-2 21237861

[B83] Sikes R. S. Ylönen H. Ylonen H. (1998). Considerations of Optimal Litter Size in Mammals. Oikos 83, 452. 10.2307/3546673

[B84] Simons M. J. Briga M. Koetsier E. Folkertsma R. Wubs M. D. Dijkstra C. (2012). Bill Redness Is Positively Associated with Reproduction and Survival in Male and Female Zebra Finches. PLoS One 7, e40721. 10.1371/journal.pone.0040721 22808243 PMC3395645

[B85] Simons M. J. Briga M. Verhulst S. (2016). Stabilizing Survival Selection on Presenescent Expression of a Sexual Ornament Followed by a Terminal Decline. J. Evol. Biol. 29, 1368–1378. 10.1111/jeb.12877 27061923 PMC4957616

[B86] Simons M. J. Cohen A. A. Verhulst S. (2012). What Does Carotenoid-dependent Coloration Tell? Plasma Carotenoid Level Signals Immunocompetence and Oxidative Stress State in Birds-A Meta-Analysis. PLoS One 7, e43088. 10.1371/journal.pone.0043088 22905205 PMC3419220

[B87] Simons M. J. Cohen A. A. Verhulst S. (2012). What Does Carotenoid-dependent Coloration Tell? Plasma Carotenoid Level Signals Immunocompetence and Oxidative Stress State in Birds-A Meta-Analysis. PLoS One 7, e43088. 10.1371/journal.pone.0043088 22905205 PMC3419220

[B88] Simons M. J. Maia R. Leenknegt B. Verhulst S. (2014). Carotenoid-dependent Signals and the Evolution of Plasma Carotenoid Levels in Birds. Am. Nat. 184, 741–751. 10.1086/678402 25438174

[B89] Simons M. J. P. Briga M. Koetsier E. Folkertsma R. Wubs M. D. Dijkstra C. (2012). Bill Redness is Positively Associated with Reproduction and Survival in Male and Female Zebra Finches. PLoS One 7, e40721. 10.1371/journal.pone.0040721 22808243 PMC3395645

[B90] Simons M. J. P. Briga M. Leenknegt B. Verhulst S. (2014). Context-dependent Effects of Carotenoid Supplementation on Reproduction in Zebra Finches. Behav. Ecol. 25. 10.1093/beheco/aru062

[B91] Simons M. J. P. Reimert I. van der Vinne V. Hambly C. Vaanholt L. M. Speakman J. R. (2011). Ambient Temperature Shapes Reproductive Output During Pregnancy and Lactation in the Common Vole (*Microtus arvalis*): A Test of the Heat Dissipation Limit Theory. J. Exp. Biol. 214, 38–49. 10.1242/jeb.044230 21147967

[B92] Smith H. G. Kallander H. Nilsson J.-A. (1989). The Trade-off Between Offspring Number and Quality in the Great Tit *Parus major* . J. Anim. Ecol. 58, 383–401. 10.2307/4837

[B93] Stearns S. C. (1989). Trade-offs in Life-History Evolution. Funct. Ecol. 3, 259–268. 10.2307/2389364

[B94] Stevens M. Párraga C. A. Cuthill I. C. Partridge J. C. Troscianko T. S. (2007). Using Digital Photography to Study Animal Coloration. Biol. J. Linn. Soc. 90, 211–237. 10.1111/j.1095-8312.2007.00725.x

[B95] Stigell P. Miyata K. Hauta-Kasari M. (2007). Wiener Estimation Method in Estimating of Spectral Reflectance from RGB Images. Pattern Recognit. Image Anal. 17, 233–242. 10.1134/s1054661807020101

[B96] Sugrue V. J. Zoller J. A. Narayan P. Lu A. T. Ortega-Recalde O. J. Grant M. J. (2021). Castration Delays Epigenetic Aging and Feminizes DNA Methylation at Androgen-Regulated Loci. Elife 10, e64932. 10.7554/eLife.64932 34227937 PMC8260231

[B97] Svensson P. A. Wong B. B. M. (2011). Carotenoid-based Signals in Behavioural Ecology: A Review. Behaviour 148, 131–189. 10.1163/000579510x548673

[B98] Számadó S. (2011). The Cost of Honesty and the Fallacy of the Handicap Principle. Anim. Behav. 81, 3–10. 10.1016/j.anbehav.2010.08.022

[B99] Therneau T. M. Grambsch P. M. (2000). Modeling Survival Data: Extending the Cox Model. Germany: Springer New York. 10.1007/978-1-4757-3294-8

[B100] Tinbergen J. M. Daan S. (1990). Family Planning in the Great Tit (Parus Major): Optimal Clutch Size as Integration of Parent and Offspring Fitness. Behaviour 114, 161–190. 10.1163/156853990x00103

[B101] van Mullem P. J. van der Vlugt J. C. (1964). On the Age, Growth and Migration of the Anadromous Stickleback *Gasterosteus aculeatus* L. Investigated in Mixed Populations. Arch. Néerl Zool 16, 111–139. 10.1163/036551664x00031

[B102] Wedekind C. Meyer P. Frischknecht M. Niggli U. A. Pfander H. (1998). Different Carotenoids and Potential Information Content of Red Coloration of Male Tree-Spined Sticklebacks. J. Chem. Ecol. 24, 787–801. 10.1023/a:1022365315836

[B103] Williams G. C. (1957). Pleiotropy, Natural Selection, and the Evolution of Senescence. Evolution 11, 398. 10.2307/2406060

[B104] Wootton R. J. Robert J. (1984). A Functional Biology of Sticklebacks. Springer, 265.

[B105] Wright D. S. Yong L. Pierotti M. E. Mckinnon J. S. (2016). Male Red Throat Coloration, Pelvic Spine Coloration, and Courtship Behaviours in Threespine Stickleback. Evol. Ecol. Res. 17 (3), 407–418.

[B106] Yamamoto R. Palmer M. Koski H. Curtis-Joseph N. Tatar M. (2020). Mapping Drosophila Insulin Receptor Structure to the Regulation of Aging Through Analysis of Amino Acid Substitutions. bioRxiv. 10.1101/2020.06.30.180505

